# Validation of suitable endogenous control genes for quantitative PCR analysis of microRNA gene expression in a rat model of endometrial cancer

**DOI:** 10.1186/1475-2867-13-45

**Published:** 2013-05-16

**Authors:** Sanja Jurcevic, Björn Olsson, Karin Klinga-Levan

**Affiliations:** 1 Systems Biology Research Centre, Tumor Biology, School of Life Sciences, Skovde, Sweden; 2 Systems Biology Research Centre, Bioinformatics, School of Life Sciences, University of Skövde, Skovde, Sweden

**Keywords:** Endogenous control genes, microRNA, Endometrial cancer

## Abstract

**Background:**

MicroRNAs are small RNA molecules that negatively regulate gene expression by translational inhibition or mRNA cleavage. The discovery that abnormal expression of particular miRNAs contributes to human disease, including cancer, has spurred growing interest in analysing expression profiles of these molecules. Quantitative polymerase chain reaction is frequently used for quantification of miRNA expression due to its sensitivity and specificity. To minimize experimental error in this system an appropriate endogenous control gene must be chosen. An ideal endogenous control gene should be expressed at a constant level across all samples and its expression stability should be unaffected by the experimental procedure.

**Results:**

The expression and validation of candidate control genes (4.5S RNA(H) A, Y1, 4.5S RNA(H) B, snoRNA, U87 and U6) was examined in 21 rat cell lines to establish the most suitable endogenous control for miRNA analysis in a rat model of cancer. The stability of these genes was analysed using geNorm and NormFinder algorithms. U87 and snoRNA were identified as the most stable control genes, while Y1 was least stable.

**Conclusion:**

This study identified the control gene that is most suitable for normalizing the miRNA expression data in rat. That reference gene will be useful when miRNAs expression are analyzed in order to find new miRNA markers for endometrial cancer in rat.

## Background

Endometrial cancer is the most frequently diagnosed gynaecological malignancy in the western world. According to the World Cancer Research Fund (WCRF) around 288 000 women worldwide develop endometrial cancer annually and 74 000 died from this cancer in 2008 [1]. This malignancy can be divided into two different types, endometrioid adenocarcinoma (type I) and serous carcinoma (type II). Type I develops from endometrial hyperplasia and is characterized by oestrogen dependence, well differentiated cells, and good prognosis. Type II, on the other hand, develops from atrophic endometrium and is oestrogen independent, with poorly differentiated cells, and has poor prognosis. The most common type, endometrioid adenocarcinoma (EAC), accounts for approximately 75% of the reported cases [2,3]. As all cancers, EAC is a complex disease, where interactions between genes and environment play important roles in the course of the disease. Studies of such diseases are complicated due to the genetic heterogeneity and environmental diversity of the human population, and therefore inbred animal model systems are often used to minimize the genetic diversity and to control the environmental factors [4]. The use of animal models does, however, not replace research on human material; these complement each other.

Virgin females of the BDII/Han inbred rat strain spontaneously develop EAC at a frequency of more than 90% during their lifetime [5,6]. Due to the high degree of conservation between the human and rat genomes, genetic aberrations in the BDII rat can be used to predict alterations associated with EAC development in humans. The similarities in physiology and pathogenesis between rats and humans imply that the BDII rat strain is a suitable model for analysis of human endometrial cancer [7].

MicroRNAs (miRNAs) are small RNA molecules that regulate gene expression by inhibition of protein translation or by degradation of their target mRNAs. It is estimated that up to 30% of human genes are regulated by miRNAs [8], and it has been shown that the functions of the target genes include a broad range of important biological processes, such as development, differentiation, growth and metabolism [9]. As for most cancers certain miRNAs are differentially expressed in EAC compared to normal tissue (see ref. [10] for a review). However, to our knowledge, no such miRNA expression analysis has yet been done in models of EAC in rat.

Due to its sensitivity and specificity quantitative polymerase chain reaction (qPCR) is commonly used for miRNA expression analysis [11]. The data from qPCR can be analysed and presented as absolute or relative values. In absolute quantification a standard curve is used to calculate the quantity of the unknown sample, whereas relative quantification is based on the relative expression of the gene of interest compared to one or more reference genes [12]. For relative quantification methods, raw qPCR data are normalized in order to correct for variation caused by the amount of starting material, variation in reaction efficiency and sample purity. This normalization can be done using one or more stably expressed endogenous control genes. A gene that is used as a reference must have stable expression independent of sample and tissue type [13]. In most qPCR studies of miRNA expression small nuclear and/or nucleolar RNAs have been used as control genes, but there are also studies in which stably expressed miRNAs have been used. There are several reasons why small non-coding RNA genes might be well suited as controls for normalizing miRNA expression data, including their stable expression, such as the nucleotide size, and the use of identical assay chemistry [14,15]. Several human and mouse small RNAs have been tested and confirmed as suitable endogenous controls for quantification of miRNA expression levels [14], but as far as we know no such study has yet been performed for small RNAs in rat.

In this study, six endogenous control genes were selected and tested for expression stability in tissues derived from rat endometrium; five small nuclear RNAs and one small cytoplasmic RNA. The identification of suitable endogenous control genes is an important initial step in expression analysis since usage of an unstable control gene for normalization could result in misleading conclusions.

## Results

Differences between the triplicates were tested for significance by ANOVA (p < 0.05), which revealed no significant differences between the three replicates in any of the six endogenous controls. Student’s *t*-test were applied for differences in gene expression between malignant and non-/pre-malignant samples were tested for in each of the six candidate genes using the (Table 1). No significant difference in expression between malignant and non-/pre-malignant samples was detected for 4.5S RNA (H) A, 4.5S RNA (H) B, snoRNA and U87, but significant differences were found for U6 and Y1 (p < 0.05).

**Table 1:** **Statistical and stability analyses of candidate endogenous control genes by*t*-test, geNorm and NormFinder**

	*t*-test*		**geNorm**		**NormFinder**		
**Control gene**	**P value**	**Rank**	**M**	**Rank**	**SD**	**Rank**	**Total rank**
U87	0.158	3	0.797	3	0.611	1	1
snoRNA	0.937	1	0.948	4	0.662	2	1
4,5S RNA(H) A	0.194	2	0.714	2	0.886	5	3
U6	0.023	6	0.714	1	0.696	3	4
4,5S RNA(H) B	0.373	4	1.016	5	0.773	4	5
Y1	0.032	5	1.152	6	1.255	6	6

*The *t*-test refers to differences in gene expression between malignant and non-/pre-malignant samples.

In order to identify and rank the most suitable control genes the data were analysed by two different algorithms, geNorm and NormFinder. GeNorm calculates the stability values (*M*) for all candidate genes, then eliminates the candidate gene with the highest *M* and repeats this procedure until the two most stable genes are left. The analysis revealed that all genes showed *M*-values below the geNorm default limit of 1.5 (Table 1). The control genes with the lowest *M*-values and highest expression stability were U6 and 4,5S RNA (H) A, both with *M* = 0.714. U87 was the third most stably expressed gene with *M* = 0.797. NormFinder calculates a stability value (standard deviation, SD) for each gene, where a low SD represents more stable expression (Table 1). In contrast to geNorm, Normfinder identified U87 and snoRNA as the two most stable genes with SD = 0.611 and 0.662, respectively. Although the two rankings differ, both algorithms identified Y1 as the most unstable gene.

**Figure 1:**
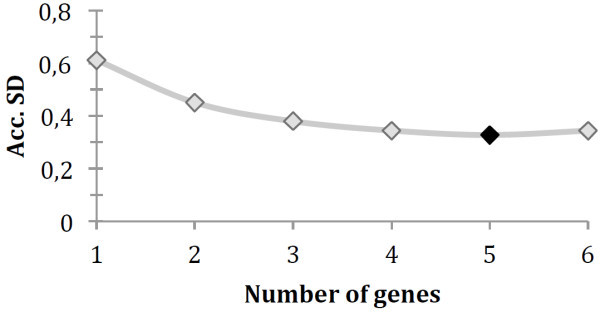
Accumulated SD using NormFinder, indicating five reference genes (black marker) as the optimal choice.

When more than one control gene is identified by Normfinder, this algorithm will also calculate the accumulated standard deviation. The optimal number of control genes is indicated by the lowest value for the accumulated SD. For this data the lowest value is reached when five control genes are used and all of them are nuclear RNAs, whereas the excluded gene (Y1) is cytoplasmic (Table 2, Figure 1).

**Table 2:** Panel of cell lines that were used in the present study

**Tumour**	**Genetic background**	**Pathology**
NUT6	(BDIIxBN)xBDII	EAC
NUT43	(BDIIxBN)xBDII	EAC
NUT50	(BDIIxBN)xBDII	EAC
NUT81	(BDIIxBN)xBDII	EAC
NUT128	(BDIIxBN)xBDII	EAC
NUT48	(BDIIxBN)xBDII	NME
NUT75	(BDIIxBN)xBDII	NME
NUT110	(BDIIxBN)xBDII	NME
NUT122	(BDIIxBN)xBDII	NME
NUT129	(BDIIxBN)xBDII	NME
NUT7	(BDIIxSPRD)xBDII	EAC
NUT41	(BDIIxSPRD)xBDII	EAC
NUT42	(BDIIxSPRD)xBDII	EAC
NUT47	(BDIIxSPRD)xBDII	EAC
NUT84	(BDIIxSPRD)xBDII	EAC
NUT58	(BDIIxSPRD)xBDII	NME
NUT68	(BDIIxSPRD)xBDII	NME
NUT74	(BDIIxSPRD)xBDII	NME
NUT89	(BDIIxSPRD)xBDII	NME
NUT91	(BDIIxSPRD)xBDII	NME

The tumour material was derived from cross between females from the EAC-susceptible BDII strain and males from BN or SPRD, respectively. NUT- tumour developed in the backcross (N1) progeny.

## Discussion

It has been clearly demonstrated in numerous studies that miRNAs play critical roles in the development of cancer [16,17]. The first documentation of a miRNA abnormality in cancer was reported by Croce and colleagues in 2002, who found that two miRNAs, miR-15 and miR-16, were lost or down-regulated in most patients with chronic lymphocytic leukaemia (CLL) [18]. Furthermore, altered miRNA expression profiles have been reported in other cancers, such as colon [19], breast [20] and endometrial cancer [21]. Identification of deregulated miRNAs in different cancer types, and knowledge of their functional significance, will advance our understanding of the aetiology of cancer and ultimately lead to development of valuable therapeutic tools.

Quantitative polymerase chain reaction (qPCR) is one of the most important methods used for gene expression analysis, since it is highly specific and allows quantitative detection of small changes in gene expression. One of the most critical points in determination of a reliable expression pattern is removal of non-biological (experimental) variation from true biological variation. The factors that influence the variability of qPCR expression values include the amount and quality of the starting material, the yields of the extraction process and the reaction efficiency. To deal with these factors it is important to use a suitable normalization strategy in the data analysis. The commonly used option for normalization is to use stably expressed endogenous control genes [22].

A suitable endogenous control gene should have abundant and constant expression across all samples. I would be very advantageous to identify control genes that are stably expressed independent of cell type and tissue type. Since no such control gene has been identified, it is important to validate the expression of endogenous control genes [13]. A good endogenous control for normalization of miRNA expression data must share similar properties with miRNAs, such as the size and stability of the RNA molecule. Some classes of small non-coding RNAs, not belonging to the miRNA class, are often expressed in an abundant and stable manner, making them good candidate control genes [14,15].

While many endogenous controls for miRNA expression studies have been identified in different species such as human and pigs [23,24], no well-established endogenous controls for miRNA quantification have been identified in rat. One of the endogenous control genes that we identified as a suitable in this report, the U6 snRNA, has been used as a reference gene in recently published studies on lung cancer [25].

In this study, we investigated the expression of 4.5S RNA (H) A, Y1, 4.5S RNA (H) B, snoRNA U87 and U6 in 21 rat cell lines. NormFinder and geNorm were used to find suitable control genes from this set of candidates, and both algorithms identified the same five control genes as most stably expressed, only differing in their ranking order. This is not unexpected since the two algorithms use different mathematical models. GeNorm calculates the stability value defined as the arithmetic mean of all pair-wise comparisons, whereas NormFinder estimates both the overall expression variation as well as the variation between sample groups. Therefore, the results of this study indicate that Y1 should not be used as endogenous control since it is ranked as the least stable by both algorithms. It is noteworthy that the five most stable genes in this study are all expressed in the nucleus, while Y1 is expressed in the cytoplasm. The Student’s *t*-test gives an additional clue to the stability of the endogenous control genes, where genes with the highest *p* values would be the most stable ones (Table 1).

Beside the stability values, NormFinder also calculates the accumulated standard deviation, which determines the number of reference genes that should be used for normalization. The results from NormFinder indicate that use of the five most stable genes (U6, 4,5S RNA (H) A, U87, snoRNA and 4,5S RNA (H) B) provides the best normalization. However, using five reference genes would be both time consuming and expensive as well as require a lot of starting material. It may therefore be advantageous to use snoRNA and U87, which are highly ranked by both algorithms, as well as by the *t*-test.

## Conclusion

In summary, careful selection of appropriate endogenous control genes plays a crucial role in expression studies. The candidate endogenous control genes should be validated, because a gene that is stable in one cell line or tissue type is not necessarily stable in other cell lines/tissue types. We recommend use of U87 and snoRNA as control genes in miRNA expression analysis in rat cells.

## Materials and methods

### Tumour material

In a previous study females from the EAC-susceptible BDII strain were crossed to males from two other inbred rat strains that are not prone to develop EAC, BN/Han and SPRD-*Cu3*/Han (hereafter BN and SPRD). An F1 generation was generated and the F1 progenies were either backcrossed to BDII females to generate an N1 generation, or intercrossed to produce F2 progeny. Tumour formation was monitored by palpation. In cases of suspected tumour, the animals were sacrificed and a necropsy was done. Pathological analysis showed that the majority of the tumours that developed in the N1 progeny were classified as EAC, but in some cases no malignant cells were observed in the removed cell mass. These tissue samples were classified as non-/pre-malignant endometrium [26]. Spontaneously arising tumours developed in approximately 25% of the F1, F2 and N1 offspring [27,28]. The NUT (backcross (N1) uterine tumour)specimens represent non-/pre-malignant cells (NME) from the endometrium or endometrial adenocarcinomas (EAC) developed in the backcross (N1) progeny.

In the present study, a total of 20 NUT endometrial cell lines were studied, of which 10 were classified as NME and 10 as EAC. Ten of the cell lines were derived from the (BNxBDII)xBDII crosses and ten from the (SPRDxBDII)xBDII crosses (Table 2). A rat embryo fibroblast (REF) cell culture was used as normal control.

### Cell cultures

The cell lines established from endometrial adenocarcinoma and non-/pre-malignant tissues were cultured in Dulbecco’s modified Eagle medium (DMEM) supplemented with 10% heat-inactivated fetal bovine serum, 100 IU/100 μg ml − 1 penicillin/streptomycin, L-glutamine, MEM essential and non-essential amino acids, and MEM vitamins solution. The cells were grown at 37°C in an atmosphere of 95% humidity and 5% CO_2_. The cells were harvested by trypsinization at 80–90% confluence (<1 × 10^6^ cells).

### Candidate control genes

A total of six candidate endogenous control genes were selected based on the availability of commercial primers. They are all small non-coding RNAs; five small nuclear RNAs (4.5S RNA (H) A, 4.5S RNA (H) B, snoRNA, U87 and U6) and one small cytoplasmic RNA (Y1). Details regarding the selected RNAs are listed in Table 3.

**Table 3:** Candidate control genes selected for evaluation of expression stability

**Assay name**	**Name**	**Accession no**	**Type of RNA**	**Database**
4.5S RNA(H) A	4.5S RNA(H) variant 5	AY228151	snRNA	GenBank
Y1	Y1 scRNA gene	U84683	scRNA	GenBank
4.5S RNA(H) B	4.5S RNA(H) variant 1	AY228147	snRNA	GenBank
snoRNA	E2 small nucleolar RNA gene	U64702	snRNA	GenBank
U87	small nucleolar RNA U87	AF272707	snRNA	GenBank
U6	U6 small nuclear 1 (RNU6-1)	NR_004394	snRNA	RefSeq

### RNA extraction, reverse transcription and qPCR

Total RNA including miRNA was isolated from the cell lines using a mirVana miRNA Isolation Kit (Ambion) following the manufacturer’s protocol. Quality and quantity of the RNA samples were determined in a NanoDrop ND-1000 Spectrophotometer (NanoDrop Technologies, USA). All the RNA samples had a 260/280 absorbance ratio of 2.0–2.1. Total RNA samples were converted to cDNA and aliquoted into triplicates using TaqMan microRNA reverse transcription kit and TaqMan miRNA primers (Applied Biosystems). After the RT step, the real-time PCR reactions were performed according to the manufacturer’s instructions; the reaction consisted of 1.33 μl cDNA (10 ng), 1.0 μl TaqMan Small RNA Assay (20Χ), 10 μl TaqMan Universal PCR Master Mix II and 7.67 μl nuclease-free water. All reactions were performed in triplicates, including the no-template control (NTC). The reactions were run on an Applied Biosystems 7300 Real Time PCR system with the following thermal cycles: one cycle of 95°C for 10 minutes; 40 cycles with a denaturation step at 95°C for 15 seconds and an annealing/extension step at 60°C for 60 seconds.

### Data analysis

Relative quantities for each candidate gene were calculated using the comparative Ct method. The Ct value represents the cycle number at which the fluorescence passes the defined threshold. One-way analysis of variance (ANOVA) was used to test for any significant difference among replicates (p < 0.05). Differences in gene expression between malignant and non-/pre-malignant samples were calculated by the Student’s *t*-test (p < 0.05).

GenEx software (MultiD Analyses AB, Göteborg, Sweden) was used to analyze the stability of candidate genes with the geNorm and NormFinder algorithms. GeNorm calculates, for each candidate control gene, the pairwise variation with all other candidate genes as the standard deviation of the log-transformed expression ratios, followed by calculation of the *M*-value. The *M*-value describes the average pairwise variation for a particular control gene with all other candidate genes. At each comparison the gene that shows largest variation to all the genes is eliminated. The process is repeated until there is only one pair of genes left. These last two genes are recommended as the optimum control genes [29].

NormFinder calculates the variance of gene expression both within groups and between groups (such as normal vs cancer). The intra- and intergroup variances are combined into a single stability value and the algorithm ranks the candidate control genes according to this combined variance. The main differences between geNorm and NormFinder are that NormFinder takes into account both the inter- and intragroup variation [30].

## Abbreviations

miRNA: microRNA; EAC: Endometrioid adenocarcinoma; NME: Non-/pre-malignant cells; qPCR: Quantitative polymerase chain reaction.

## Competing interests

The authors declare that they have no competing interests.

## Authors’ contributions

SJ performed all the experiments and data analysis and helped to draft the manuscript. KKL and BO participated in the analysis of data as well as helped to draft the manuscript. All authors read and approved the final manuscript.
